# A Never-Ending Challenge: Intestinal Tuberculosis or Inflammatory Bowel Disease

**DOI:** 10.7759/cureus.16282

**Published:** 2021-07-09

**Authors:** Arda Yavuz, Ayşe Nur Toksöz Yıldırım, Kübra Akan, İlyas Tuncer

**Affiliations:** 1 Gastroenterology and Hepatology, Istanbul Medeniyet University Göztepe Research and Training Hospital, Istanbul, TUR; 2 Pathology, Istanbul Medeniyet University Göztepe Research and Training Hospital, Istanbul, TUR

**Keywords:** tuberculosis, crohn’s disease, inflammatory bowel disease, laparascopy, colonoscopy

## Abstract

Intestinal tuberculosis is an uncommon form of tuberculosis, and its diagnosis remains a challenge in patients with Crohn's disease. The clinical, endoscopic, radiologic, and histologic features of Crohn's disease and tuberculosis are remarkably similar, posing a diagnostic challenge. Accurate diagnosis of these two conditions remains vital to the decision on the treatment of the patients. Computerized tomography, endoscopic ultrasound (EUS), capsule endoscopy, balloon enteroscopy, ascitic fluid adenosine deaminase (ADA), tuberculosis polymerase chain reaction (TB-PCR), GeneXpert MTB/RIF assay (Cepheid, Sunnyvale, CA), and laparoscopy can be beneficial in the diagnosis of intestinal tuberculosis. Herein, we report a case where tuberculosis could not be documented, although the patient displayed lymphocytosis in ascites and weight loss. Laparoscopy was diagnostic and the patient benefited from the correct treatment.

## Introduction

Tuberculosis infection mainly presents as pulmonary infection, but in 10-20% of cases, extrapulmonary involvement is also observed [1]. The incidence of tuberculosis is higher in immunosuppressed patients who are infected with the human immunodeficiency virus or are receiving chemotherapy in developed countries. Further, the incidence of tuberculosis in developing countries is also high due to a greater prevalence of inadequate sanitation and poor living conditions.

Intestinal tuberculosis can present as chronic diarrhea, weight loss, fever, and ascites [2]. Differential diagnoses include Crohn's disease, malignancies, periappendiceal abscesses, yersinia, or an entameba infection. Differing sensitivity and specificity of diagnostic methods can lead to combining methods, repeated biopsies, and in some cases, laparoscopy. In cases of high clinical suspicion, although the bacillus cannot be detected, treatment may be initiated [3].

Herein, we report a case with a high clinical suspicion of tuberculosis. Crohn's disease and malignancy were considered as differential diagnoses based on the patient's initial evaluation. The patient had mass images on computed tomography (CT), weight loss, and narrowing of the right colon. Ascites tap directed us to laparoscopy, in which small whitish tuberculomas were observed. The patient then started anti-tuberculosis treatment.

## Case presentation

**Figure 1:**
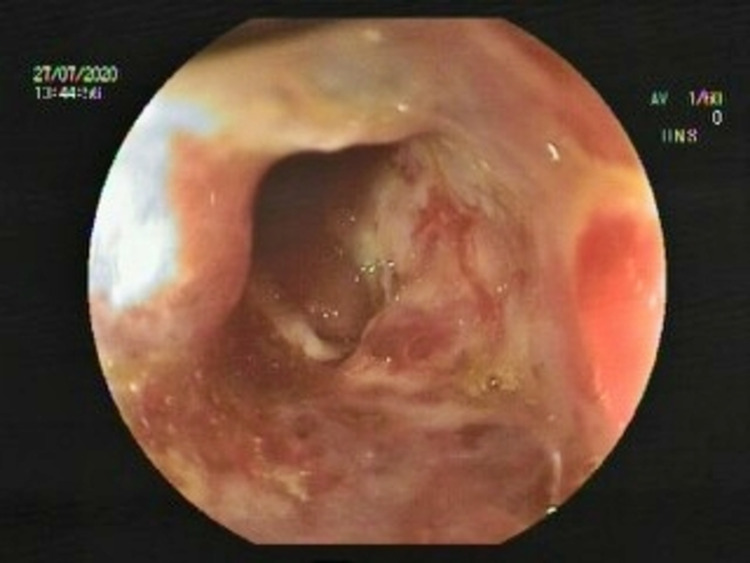
Ulcero-vegetan mass image in the right colon

A 40-year-old male patient with a 1.5-year history of right colon dominant Crohn’s disease was admitted to the hospital with stomachache and subfebrile fever. He had six to seven loose stools a day without bleeding. His vital parameters were normal, and his bowel sounds were hyperactive. The patient had lost approximately 6-7 kg in body mass. The patient had no relevant family history and the chest radiograph was normal. During colonoscopy, which was performed in another endoscopy unit in 2019, an ulcero-vegetan mass was observed narrowing the right colon. Subsequent biopsy showed granuloma. Furthermore, intestinal tuberculosis was excluded. Due to the patient’s pain and inadequate treatment response, he was accepted to the clinic for further evaluation. He was taking mesalazine as a medication. Ulcero-vegetan mass image in the right colon was observed in colonoscopy (Figure 1).

**Figure 2:**
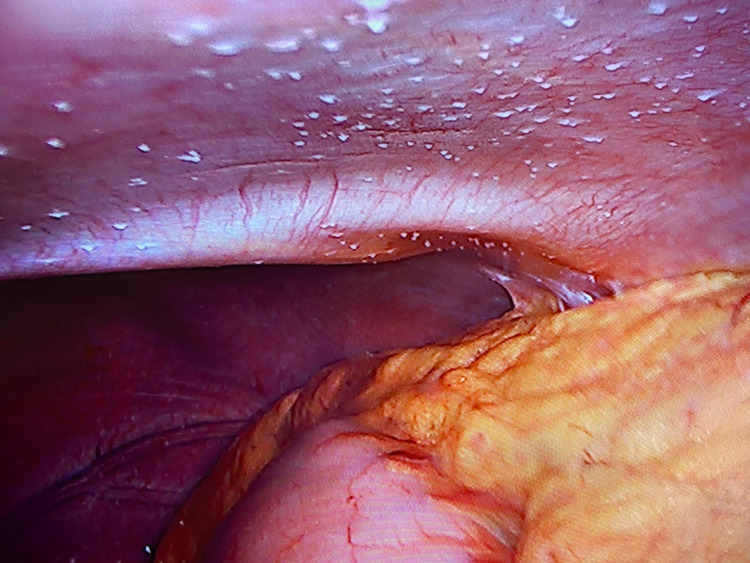
Small white tuberculomas in laparoscopy

**Figure 3:**
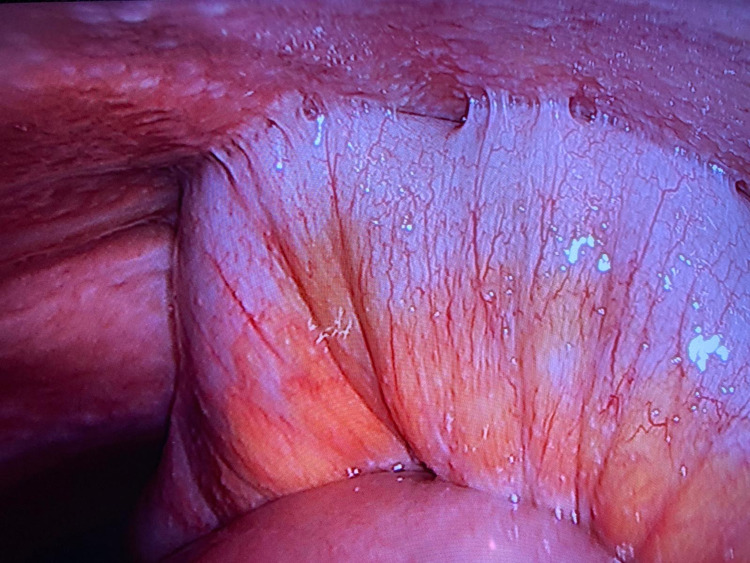
Violin string fibrinous strands

Abdominal ultrasonography showed ascites in the abdomen. Ascitic tap was performed, reporting serum-ascites albumin gradient (SAAG) as <1.1 g/dL, white blood cell (WBC) count in ascites fluid was 5011 cell/mm^3^, and lymphocyte ratio was 88.4%. The CA-125 level was 369.7 IU/mL. Due to lymphocytosis in the ascites fluid, laparoscopy was performed, showing whitish tuberculomas and violin string fibrinous strands (Figures 2, 3).

**Figure 4:**
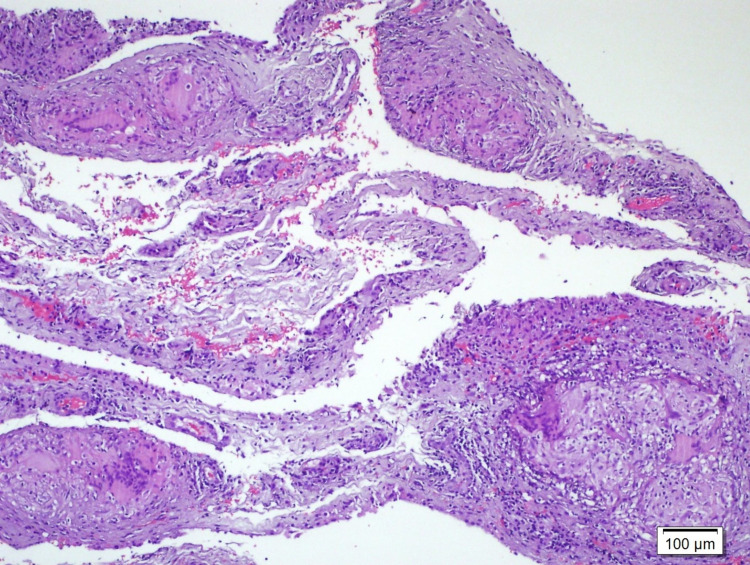
"Langhans" type multinuclear giant cells and lymphocytes (hematoxylin and eosin × 100) in granulomas consisting of epithelioid histiocytes

**Figure 5:**
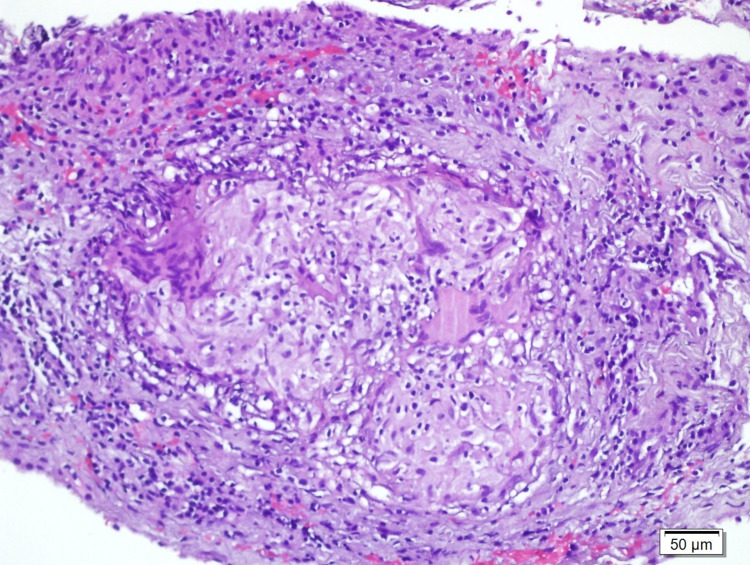
"Langhans" type multinuclear giant cells and lymphocytes (hematoxylin and eosin × 200) in granulomas consisting of epithelioid histiocytes

Histological examination of serial sections revealed non-necrotizing granuloma formation. "Langhans" type giant cells were seen embedded in the fibrous stroma or in the center of the granuloma. In the immunohistochemical examination, CD 68 was positive in histiocytes. In a histochemical examination, no bacilli were seen with Ziehl-Neelsen staining (Figures 4, 5).

**Figure 6:**
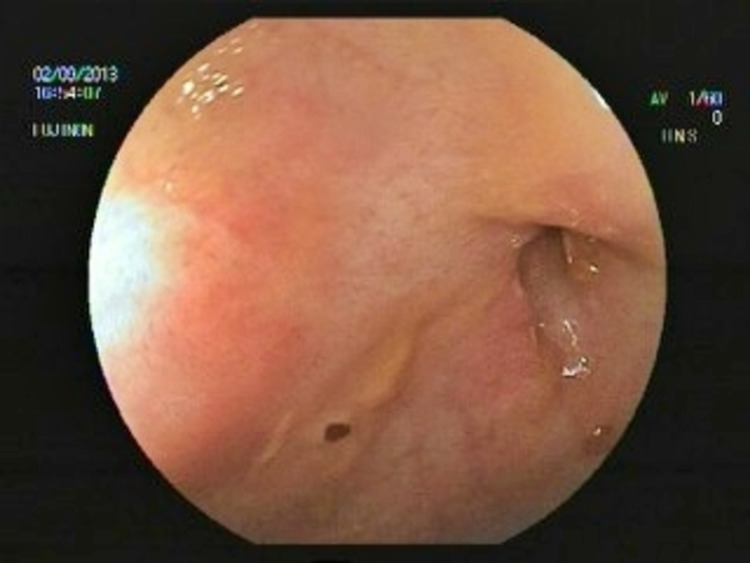
Stricture in the right colon after treatment

As a result of the small whitish tuberculum, the patient was diagnosed with intestinal tuberculosis, and anti-tuberculosis treatment was initiated. Clinical improvement was observed within two weeks. After 10 months of treatment, a control colonoscopy was performed, and the mucosa had completely healed (Figure 6).

Although the stricture had not resolved, the patient’s symptoms had eased substantially and he had regained 6 kg in body mass. However, the patient reported periods of stomachache and therefore consulted for surgery.

## Discussion

Intestinal tuberculosis presents with abdominal pain, fever, weight loss, nausea, diarrhea or constipation, ascites, and, more rarely, hepatomegaly and splenomegaly. Our patient had subfebrile fever, night sweats, weight loss, diarrhea, and ascites. Intestinal obstruction also occurs in approximately 5% of intestinal tuberculosis cases. Obtaining a family history is essential; however, our patient did not have a family history of tuberculosis.

An ascites tap can be helpful for diagnosis. Patients with tuberculosis peritonitis usually have lymphocytic ascites with SAAG <1.1 g/dL and a cell count of 150-4000 cells/mL [4]. The sensitivity of acid-fast bacillus (AFB) smears and mycobacterial cultures is low, but ascites fluid ADA level is a valuable tool, and elevated (30-39 IU/L) levels support the diagnosis of intestinal tuberculosis [5]. Peritoneal fluid interferon-gamma level may also be a helpful tool, but this has not yet been approved by the United States Food and Drug Administration (FDA) for intestinal tuberculosis diagnosis. On CT imaging, circumferential wall thickening of the terminal ileum and cecum, asymmetric thickening of the ileocecal valve, mesenteric lymphadenopathy with central low attenuation areas, and involvement of other organs can aid diagnosis.

Endoscopic findings of intestinal tuberculosis include transverse ulcers, hypertrophic mucosa, scaring, fibrous bands, inflammatory polyps, destruction of the ileocecal valve, and hyperemic nodules, but none of these are specific findings. Endoscopic ultrasound (EUS)-guided fine-needle aspiration may be effective for histopathologic diagnosis when there is evidence of lymphadenopathy or solid organ involvement. Capsule endoscopy can also provide additional information. Ileocecal valve involvement is more common in intestinal tuberculosis, while aphthous ulcers and multi-segment involvement of the small bowel are more common in Crohn's disease [6]. Small bowel enteroscopy can also provide an opportunity for biopsy. In certain cases, endoscopy is not sufficient for diagnosis, as in this case, and laparoscopy can be lifesaving. A thickened peritoneum with yellowish-white lesions with or without adhesions, thickened peritoneum with or without adhesions, and fibroadhesive pattern are the three major patterns that can be seen in laparoscopy for tuberculosis peritonitis. Enlarged lymph nodes, violin string fibrinous strands, and omental thickening can also be seen [7]. Blind peritoneal biopsies have a low success rate.

At histology, caseating granulomas or positive acid-fast bacilli staining, confluent (≥5 /biopsy) and large (diameter >200 µm) granulomas, submucosal granulomas, ulcers lined by epithelioid histiocytes, and disproportionate submucosal inflammation are suggestive of tuberculosis [8]. In cases of high clinical suspicion, although the bacillus cannot be detected, treatment may be initiated. Patients with intestinal tuberculosis should receive quadruple anti-tuberculosis treatment. In general, there is no precise timing for control colonoscopy.

## Conclusions

Abdominal tuberculosis is clinically challenging due to its nonspecific symptomatology, difficulty in isolation of the bacillus, and different sensitivities of the diagnostic approaches. Clinical suspicion and family history are the critical cornerstones of diagnosis. In this case report, we would like to emphasize the importance of laparoscopy.
